# Weight loss is associated with sustained improvement of disease activity and cardiovascular risk factors in patients with psoriatic arthritis and obesity: a prospective intervention study with two years of follow-up

**DOI:** 10.1186/s13075-020-02350-5

**Published:** 2020-10-22

**Authors:** Eva Klingberg, Sofia Björkman, Björn Eliasson, Ingrid Larsson, Annelie Bilberg

**Affiliations:** 1 Department of Rheumatology and Inflammation Research, Sahlgrenska Academy at the University of Gothenburg, Gothenburg, Sweden; 2 Department of Rheumatology at Sahlgrenska University Hospital, Gröna stråket 14, SE-41345 Gothenburg, Sweden; 3 Department of Gastroenterology and Hepatology, Sahlgrenska University Hospital, Gothenburg, Institute of Medicine, Sahlgrenska Academy at University of Gothenburg, Gothenburg, Sweden; 4 Department of Medicine, Institute of Medicine, Sahlgrenska University Hospital, University of Gothenburg, Gothenburg, Sweden; 5 Institute of Neuroscience and Physiology, Section of Health and Rehabilitation, Physiotherapy, Sahlgrenska Academy at the University of Gothenburg, Gothenburg, Sweden

**Keywords:** Psoriatic arthritis, Psoriasis, Obesity, Metabolic syndrome, Weight loss, VLED, Cardiovascular disease

## Abstract

**Background:**

Obesity is overrepresented in patients with psoriatic arthritis (PsA) and associated with increased disease activity. We have previously shown in 41 patients with PsA (Caspar criteria) and obesity (body mass index; BMI ≥33 kg/m^2^) that weight loss treatment with Very Low Energy Liquid Diet (VLED), 640 kcal/day during 12–16 weeks, followed by a structured reintroduction of an energy restricted diet resulted in a median weight loss of 18.6% and concomitantly a significant improvement of the disease activity in joints, entheses and skin.

The objectives of this follow-up were to study the effects of the weight loss treatment on disease activity in longer term (12 and 24 months) and to study the effects on cardiovascular risk factors.

**Methods:**

The patients were assessed with 66/68 joints count, Leeds enthesitis index (LEI), body surface area, blood pressure, BMI, questionnaires and fasting blood samples at the 12- and 24-month visits.

**Results:**

In total, 39 and 35 PsA patients attended the 12- and the 24-month visits, respectively. Median weight loss since baseline was 16.0% (IQR 10.5–22.4) and 7.4% (IQR 5.1–14.0) at the 12- and 24-months follow-up. The 66/68 swollen/tender joints score, LEI, CRP and HAQ score were still significantly reduced at the 12- and 24-month visits compared to baseline. The number of patients with Minimal Disease Activity increased from 28.2% (11/39) at baseline, to 38.5% (15/39; *p* = 0.008) and 45.7% (16/35; *p* = 0.016) at the 12- and 24-month visits.

The weight loss was also associated with improved levels of serum lipids, glucose and urate and the antihypertensive treatment was reduced or stopped in five patients during the follow-up.

**Conclusions:**

Weight loss treatment, with VLED included in the program, was associated with long-term improvement of measures of disease activity, self-reported function and markers of the metabolic syndrome after 24-months follow-up.

**Trial registration:**

ClinicalTrials.gov identifier: NCT02917434, Registered September 28, 2016- Retrospectively registered.

## Background

Psoriatic arthritis (PsA) is an inflammatory disease characterized by psoriasis, arthritis, enthesitis and dactylitis [1]. Obesity, diabetes, hypertension, hyperlipidemia and the metabolic syndrome (MetS) are overrepresented in both psoriasis and PsA [2–4] and the patients are at increased risk of cardiovascular morbidity [5–9]. Studies indicate that obesity may play a pathophysiologic role in psoriasis and PsA, since obesity is associated with an increased risk of developing these conditions [10–13]. Obesity is also associated with increased disease activity [14, 15] and poorer effect of treatment [16–19]. Although several studies have shown a relation between obesity and disease activity, there is a lack of studies investigating the effects of weight loss in PsA.

We have previously in an open interventional study shown that weight loss treatment with Very Low Energy Diet (VLED) in 41 patients with PsA and obesity resulted in a substantial weight loss of median 18.7 kg (interquartile range: IQR 14.6–26.5) after six months, which was associated with positive effects on the disease activity in joints, entheses and skin [20]. Significant improvements were noted regarding number of swollen and tender joints, enthesitis, extent of psoriasis, C-reactive protein (CRP), pain, fatigue and physical function at six months observation. The proportion of patients with Minimal Disease Activity (MDA) increased from 29% to 54% (*p* = 0.002) [20].

Short term weight loss can however easily be regained. Moreover, the anti-inflammatory effects seen in our previous study could have been caused by, both metabolic effects due to energy restriction during the initial phase of rapid weight loss, as well as loss of adipose tissue and lessened production of pro-inflammatory cytokines and adipokines therein.

The primary aim of this follow-up was to study the association between weight loss and disease activity in longer term; 12 and 24 months in patients with PsA and obesity. A secondary aim was to study the effects of weight loss on aspects of the MetS in patients with PsA and matched controls.

## Methods

### Patients and controls

The patients were recruited from the departments of Rheumatology at Sahlgrenska University Hospital and the hospitals of Borås and Alingsås. Inclusion criteria were having PsA fulfilling the Classification for Psoriatic Arthritis (CASPAR) criteria, a body mass index (BMI) of 33 kg/m^2^ or more, and age 25–75 years [21]. If treated with conventional synthetic and/or biologic Disease-Modifying Anti-Rheumatic Drugs (cs and/or bDMARDs), the treatment doses had to be stable and unchanged from three months prior to baseline until six months after baseline. After six months the patients were allowed to change immunomodulating therapy. Exclusion criteria were pregnancy, porphyria, epilepsy, type 1 diabetes, severe heart, kidney or catabolic disease, binge eating disorder, treatment with warfarin, lithium or phenytoin, mental imbalance affecting participation, a history of a myocardial infarction, stroke, major surgery or trauma during the last three months, or being treated for cancer during the last five years.

The controls were patients with obesity planned for treatment with VLED and matched for sex, age and body weight to the PsA patients, and they were recruited from the Regional Obesity Centre of the Region Western Sweden at Sahlgrenska University Hospital. Exclusion criteria for controls were having psoriasis, PsA or any other inflammatory rheumatic disease in addition to the above stated exclusion criteria for the PsA patients. The controls were used as comparators for vital signs, biochemical measures and effects on cardiovascular risk factors of the VLED treatment.

The partcipants´ treatments for hypertension, diabetes and hyperlipidemia were monitored and could be changed throughout the duration of the study.

All the participants in the study gave their written informed consent. The study was approved by the Regional Ethics Committee in Gothenburg and carried out in accordance with the Helsinki declaration.

### The intervention: weight loss treatment with very low energy diet (VLED)

Weight loss treatment with VLED was provided at the Regional Obesity Centre of the Region Western Sweden at Sahlgrenska University Hospital, within a framework of medical follow-up, dietary advice and support during 12 months. The VLED provides a mixture of carbohydrates, proteins and fats, with added recommended doses of vitamins, minerals, trace elements and essential fatty acids, in powder formulas that require mixing with hot or cold water before consumption as shakes or soups. The VLED treatment in our study consisted of four doses of VLED per day, providing a daily intake of 640 kcal. (Cambridge Weight Plan Limited, Corby, UK). Depending on baseline BMI, < 40 or ≥ 40 kg/m^2^, the strict VLED treatment was maintained during 12 or 16 weeks. Non-energy-containing beverages were allowed ad libitum. After the strict VLED period, food was gradually reintroduced during a period of 12 weeks and each participant was given a personal dietary advise based on individual energy requirements for weight stability with a reduction of 30% to achieve further weight loss. The macronutrient distribution in the recommended diet was approximately 20 energy percent (E%) protein, 30–35 E% fat and 45–50 E% carbohydrates. Each patient was given a written meal plan including three main meals and a snack meal. The dietary advice focus on healthy food selection including vegetables, fruits, whole-grain cereals, low-fat dairy products and vegetable fats and oils, fish, poultry and lean meat and limited amount of energy-dense and nutrient-sparse foods such as chocolate, sweets, bakery foods, sugar-sweetened beverages. The food selection was modified based on individual food preferences for each patient. The overall aim of the dietary advice was to restrict total energy intake, saturated fats and added sugar and increase dietary fiber, unsaturated fats as well as essential nutrients. After 12 months the patients were no longer followed at the Regional Obesity Centre, but at the departments of Rheumatology. In addition, all patients were seen by a physiotherapist at baseline and after six, 12 and 24 months and were instructed to engage in health-enhancing physical activity at least 150 min per week.

### Measures of assessment

The patients with PsA were assessed at baseline and after three, six, 12 and 24 months. Body height was measured at baseline, and weight was measured at baseline and at every follow-up and BMI was calculated. Waist circumference was measured in standing position with a tape measure midway between the lower rib and iliac crest. Joints were examined with 66/68 swollen/tender joints count and entheses with Leeds enthesitis index [22]. The extent of psoriasis was evaluated with Body Surface Area (BSA) [23]. Quality of life related to psoriasis was assessed with the Dermatology Life Quality Index (DLQI) [24]. The patients’ experience of global disease activity, pain and fatigue and the physician’s evaluation of the patients’ global disease activity was assessed with Visual Analogue Scales (VAS). Activity limitations and function were assessed using the Health Assessment Questionnaire (HAQ) and Bath Ankylosing Spondylitis Functional Index (BASFI) [25, 26]. Both the Disease Activity Score using 28 joint counts based on CRP (DAS28CRP) and the Disease Activity in PSoriatic Arthritis (DAPSA) score were calculated [27, 28]. Minimal disease activity (MDA) was defined as meeting five of the seven following criteria: tender joint count 68 ≤ 1, swollen joint count 66 ≤ 1, psoriasis BSA ≤ 3%, patient pain VAS ≤ 15 mm, patient global disease activity VAS ≤ 20 mm, HAQ ≤ 0.5 and tender entheseal points ≤ 1 [29].

At the 24 month visit the questionnaire Patient Global Impression of Change (PGIC) was used, where patients are asked to rate their overall status from the start of the study using the following scale: (1) very much improved, (2) much improved (3) minimally improved, (4) no change, (5) minimally worse, (6) much worse, (7) very much worse [30].

The controls were assessed at baseline and at three, six and 12 months, but not at 24 months, with measurements of height, weight, waist circumference and blood pressure. Information about medical history and current medication was obtained via questionnaires.

Blood samples were drawn from the participants in the morning after ≥8 h of fasting and analyzed for hemoglobin (Hb), white blood cell count (WBC), platelet count (PLT), C-reactive protein (CRP), alanine transaminase (ALT), creatinine, urate, glucose, glycosylated hemoglobin (HbA1c), total cholesterol (TC), high-density lipoprotein (HDL) cholesterol, low-density lipoprotein (LDL) cholesterol and triglycerides (TG) using standard laboratory techniques at Sahlgrenska University Hospital.

Occurrence of MetS was defined as exhibiting three or more of the following five criteria [31]: ^1^elevated waist circumference (≥80 cm for women and ≥ 94 cm for men), ^2^elevated TG (TG ≥1.7 mmol/L or drug treatment for elevated TG), ^3^reduced HDL (HDL < 1.3 mmol/L for women and < 1.0 mmol/L for men or drug treatment for reduced HDL), ^4^elevated blood pressure (BP) (systolic BP ≥130 mmHg or diastolic BP ≥85 mmHg or antihypertensive drug treatment), ^5^elevated fasting glucose (plasma glucose ≥5,6 mmol/L or previously diagnosed type 2 diabetes).

### Statistical analyses

Statistical analyses were made using SPSS Statistics version 25 (IBM, Chicago, USA). Descriptive statistics are presented as median and IQR. Wilcoxon Signed Rank Test was used to compare continuous related samples and McNemar’s test to compare categorical related samples. Correlations were calculated using Spearman’s correlation (r_S_). All tests were two-tailed and *p* ≤ 0.05 was considered statistically significant. Only the 39 PsA patients and 39 controls who attended the 12- month visit and the 35 PsA patients who attended the 24-month visit, were included in the statistical analyses.

## Results

### Characteristics of the study population

**Fig. 1:**
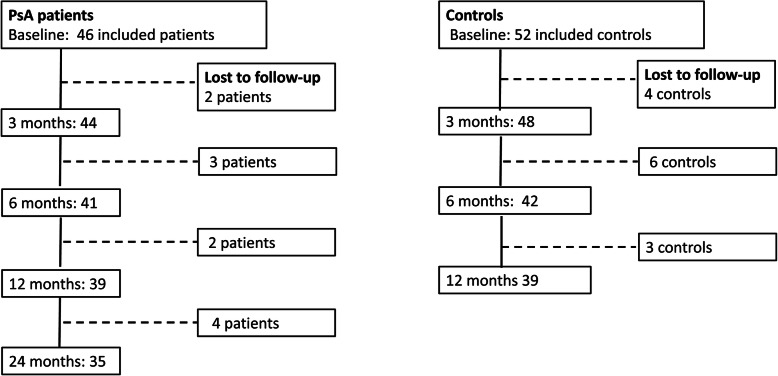
Flow chart for the study from baseline to 24 months showing participation and participants lost to follow-up

Included participants and those lost to follow-up are shown in Fig. 1.

In total, 46 patients with PsA were included and started VLED treatment, whereof 41, 39 and 35 patients attended the six, 12- and 24-month visits respectively. The 11 patients (8 women and 3 men) lost to follow-up during the whole study were significantly younger than the patients who continued in the study (*p* = 0.005). The demographics and the medication of the completers are shown in Table 1. Treatment with cs/bDMARDs was unchanged compared to baseline in 89% (31/35) at the 12-month visit and in 74% (26/35) at the 24-month visit.

**Table 1:** Age, sex and medication of the patients with psoriatic arthritis (PsA) at baseline (BL) and the 12- months (M12) and 24-months (M24) visits

	BL (N = 39)	M12 (N = 39)	M24 (*N* = 35)
**Women,** n(%)		25 (64.1)	21 (60.0)
**Men**, n(%)		14 (35.9)	14 (40.0)
**Age**, years (IQR)		56 (49–63)	59 (51–56)
**NSAIDs**, n (%)	27 (69.2)	25 (64.1)	21 (60.0)
**TNFi all**, n (%)	15 (38.5)	16 (41.0)	14 (40.0)
**TNFi in monotherapy**	4 (10.3)	4 (10.3)	3 (8.6)
**TNFi + csDMARD**	11 (28.2)	12 (30.7)	11 (31.4)
**Ustekinumab monotherapy**, n (%)	1 (2.6)	0	0
**Secukinumab monotherapy**, n (%)	0	1 (2.6)	2 (5.7)
**csDMARD without biologic**, n (%)	17 (43.6)	16 (41.0)	14 (40.0)
**Methotrexate**	11 (28.2)	11 (28.3)	10 (28.6)
**Sulfasalazine**	2 (5.1)	1 (2.6)	1 (2.9)
**Apremilast**	1 (2.6)	1 (2.6)	2 (5.7)
**Methotrexate + Sulfasalazine**	3 (7.7)	3 (7.7)	1 (2.9)
**Prednisolone**, n (%)	3 (7.7)	2 (5.1)	2 (5.7)
**Anti-hypertensives**, n (%)	17 (43.6)	14 (35.9)	14 (40.0)
**Lipid lowering therapy**, n (%)	6 (15.4)	5 (12.8)	5 (14.3)
**Oral anti-diabetics**, n (%)	1 (2.6)	2 (5.1)	2 (5.7)
**Allopurinol**, n (%)	2 (5.1)	2 (5.1)	2 (5.7)

csDMARD = conventional synthetic Disease Modifying Anti-Rheumatic Drug, IQR = inter-quartile range, NSAID = non-steroidal anti-inflammatory drug, TNFi = tumor necrosis factor inhibitor

A total of 52 controls were included and started VLED treatment, whereof 42 and 39 controls came to the six- and 12-months visits respectively. A comparison of baseline characteristics between the PsA patients and controls is shown in Table 2.

**Table 2:** Comparison between PsA patients and controls at baseline

	Patients N = 39	Controls N = 39	***p***-value
**Women,** n(%)	25 (64.1)	29 (74.4)	0.462
**Men**, n(%)	14 (35.9)	10 (25.6)	
**Age** years	55 (48–62)	56 (48–60)	0.617
**Body weight** kg	106.0 (93.5–112.5	107.0 (96.5–122.2)	0.358
**Body height cm**	168.0 (161.0–176.0)	165.0 (161.0–170.0)	0.180
**BMI** kg/m^2^	35.2 (33.9–37.9)	38.5 (36.9–41.7)	**< 0.001**
**Waistline** cm	115.0 (112.0–122.0)	117.0 (107–126.5)	0.635
**BP systolic** mmHg	127.5 (115.0–136.0)	122.0 (115.0–137.0)	0.503
**BP diastol** mmHg	77.5 (70.0–82.5)	77.0 (70.0–85.0)	0.736
**CRP** mg/L	5 (3–9)	4 (2–6)	0.137
**Hb** g/L	144 (131–150)	141 (135–149)	0.881
**WBC** 10^9^/L	6.1 (5.3–7.7)	6.6 (5.6–7.6)	0.719
**PLT** 10^9^/L	270 (202–299)	266 (225–302)	0.924
**S-TC** mmol/L	5.5 (4.6–6.2)	4.9 (4.1–5.6)	**0.034**
**S-LDL** mmol/L	3.6 (3.0–4.4)	3.2 (2.54.0)	0.08
**S- HDL** mmol/L	1.4 (1.1–1.6)	1.4 (1.0–1.6)	0.867
**S- TG** mmol/L	1.6 (1.2–2.4)	1.7 (1.2–2.1)	0.512
**HbA1c** mmol/mol	35.5 (32.0–37.2)	37.5 (35.0–44.0)	**0.003**
**S-glucose** mmol/L	5.7 (5.3–6.2)	6.2 (5.8–7.1)	**0.003**
**Creatinine** μmol/L	75 (64–84)	68 (62–79)	0.240
**S-urate** μmol/L	330 (280–400)	350 (283–384)	0.928
**ALT** μkat/L	0.52 (0.41–0.68)	0.50 (0.42–0.68)	0.806
**Anti-hypertensives**, n (%)	17 (43.6)	16 (41.0)	0.819
**Lipid lowering therapy** n (%)	6 (15.4)	11 (28.2)	0.170
**Oral anti-diabetics** n (%)	1 (2.6)	4 (10.3)	0.358
**Insulin** n (%)	0 (0)	1 (2.6)	1.00
**Allopurinol**, n (%)	2 (5.1)	1 (2.6)	1.00
**Mets** n (%)	30 (76.9)	28 (71.8)	0.614

ALT = alanine transaminase, BMI = Body Mass Index, BP = blood pressure, CRP = C- reactive protein, Hb = hemoglobin HbA1c = glycosylated hemoglobin, HDL = high-density lipoprotein cholesterol, LDL = low-density lipoprotein cholesterol, PLT = platelet count, S– =serum, TC = total cholesterol, TG = triglycerides, Mets = metabolic syndrome, WBC = White Blood Cell count

### Body weight and disease activity after 12 months

There were 39 patients with PsA who attended the 12-month visit. At baseline their median weight was 106.0 (IQR 93.5–112.5) kg and at 12 months 87.5 (80.6–95.5) kg (*p* < 0.001). In median, the patients had lost 16.1 (10.5–22.8) % of their baseline weight. In total, 77% (*N* = 30/39) of the patients had a weight loss of 10% or more compared to baseline, and 17.9% (*N* = 7/39) a weight loss of 5–10%. The majority of patients (34/39) had however regained some weight between the six- and 12-month visits, in median 3.9 (IQR 1.5–6.6) kg.

**Fig. 2:**
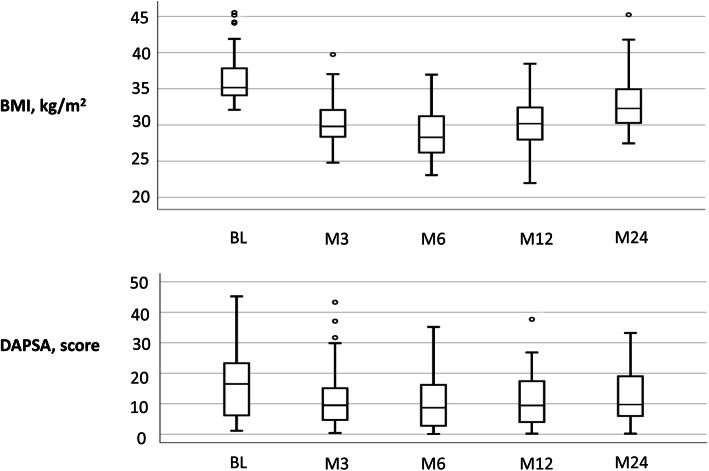
Boxplots showing the distribution of body mass index (BMI) and Disease Activity in PSoriatic Arthritis (DAPSA) scores at baseline and the visits at three, six, 12 and 24 months

A majority of the disease activity variables were still significantly improved at the 12-month follow-up compared to baseline, including swollen/66 and tender/68 joints count, Leeds enthesitis index, BSA, CRP, VAS for global health and fatigue, HAQ, BASFI, DAS28-CRP and DAPSA. Criteria for MDA was met by 28.2% (11/39) at baseline and 38.5% (15/39) at the 12-months follow-up (*p* = 0.008). However, no significant difference was found in VAS pain and DLQI at 12 months follow-up, compared to baseline. (Table 3 and Fig. 2).

**Table 3:** Body weight, BMI and measures of disease activity and function before weight loss treatment at baseline (BL) and after 12-months (M12) and 24-months (M24) in patients with psoriatic arthritis (PsA). Values are median and inter-quartile range (IQR)

	BL N = 39	M12 N = 39	M24 N = 35	BL vs M12 p-value N = 39	BL vs M24 p-value N = 35
**Body weight** kg	106.0 (93.5–112.5)	87.5 (80.6–95.5)	92.7 (85.7–100.7)	**< 0.001**	**< 0.001**
**BMI** kg/m^2^	35.2 (33.9–37.9)	30.5 (28.0–32.9)	32.3 (30.3–35.4)	**< 0.001**	**< 0.001**
**Tender joints 68** count	4 (1–14)	3 (0–6)	2 (0–7)	**0.001**	**< 0.001**
**Swollen joints 66** count	0 (0–1)	0 (0–1)	0 (0–0)	**0.015**	**0.003**
**Leeds enthesitis index**, count	1 (0–4)	0 (0–2)	1 (0–3)	**< 0.001**	**0.002**
**BSA** %	0.75 (0–2)	0.25 (0–1)	0.25 (0–1.5)	**0.018**	0.194
**CRP** mg/L	5 (3–9)	2 (1–5)	3 (2–5)	**0.009**	**0.011**
**Hb** g/L	144 (131–150)	143 (131–150)	142 (135–149)	0.296	0.537
**WBC** 10^9^/L	6.1 (5.3–7.7)	5.7 (5.0–7.4)	5.9 (4.7–7.8)	0.133	0.346
**PLT** 10^9^/L	270 (202–299)	262 (203–308)	273 (220–332)	0.368	0.334
**VAS Pain** mm	35 (15–60)	30 (14–46)	35 (15–55)	0.073	0.799
**VAS Global health**, mm	40 (20–70)	30 (17–52)	40 (20–60)	**0.023**	0.282
**VAS Fatigue** mm	50 (30–70)	30 (20–60)	60 (30–80)	**0.021**	0.064
**HAQ** score	0.63 (0.13–1.00)	0.25 (0–0.63)	0.25 (0.13–0.88)	**0.002**	**0.033**
**BASFI** score	2.6 (1.4–5.2)	1.6 (0.57–3.6)	2.1 (0.9–4.2)	**< 0.001**	0.131
**DLQI** score	1 (0–4)	1 (0–4)	1 (0–4)	0.380	0.982
**DAS-28CRP** score	3.1 (2.1–3.7)	2.4 (1.8–3.3)	2.4 (1.7–2.9)	**< 0.001**	**< 0.001**
**DAPSA** score	16.1 (6.2–23.3)	9.5 (4.8–18.1)	9.7 (5.3–19.0)	**< 0.001**	**< 0.001**
**MDA** n (%)	11 (28.2)	15 (38.5)	16 (45.7)	**0.008**	**0.016**

BASFI = Bath Ankylosing Spondylitis Functional Index, BMI = Body Mass Index, BSA = Body Surface Area, CRP = C- reactive protein, DAPSA = Disease Activity in PSoriatic Arthritis, DAS28CRP = Disease Activity Score using 28 joint counts based on CRP, DLQI = Dermatology Life Quality Index, ESR = erythrocyte sedimentation rate, HAQ = Health Assessment Questionnaire, Hb = hemoglobin, MDA = Minimal Disease Activity, PLT = platelet count, VAS = Visual Analogue Scale, WBC = White Blood Cell count

Subgroup analyses showed that the women with PsA demonstrated a greater reduction of CRP (∆CRP) compared to the men (*p* = 0.041), and the men a greater reduction in swollen joints (∆swollen/66) than the women (*p* = 0.047), but there was no significant difference in change in BMI, DAPSA, DA28CRP, BSA, HAQ between the sexes. PsA patients on a bDMARD had a greater reduction of tender joints (∆tender/68; *p* = 0.015) than those without, but otherwise the change in disease activity was similar between the groups at 12 months. The weight loss and change in CRP was also comparable in the PsA patients and controls at the six and 12-month visits (Table 4).

**Table 4:** Comparison between PsA patients and controls: change in bodyweight, BMI, CRP and urate at the 6-months (M6) and 12-months (M12) visits

	Patients N = 39	Controls N = 39	p-value
**M6**			
**Weight loss since baseline**. kg	19.0 (15.6–27.0)	23.0 (17.5–29.5)	0.280
**Weight loss/baseline weight** %	18.7 (15.2–26.7)	21.7 (15.5–26.4)	0.600
**∆ BMI** kg/m^2^	7.1 (5.3–9.6]	8.0 (6.3–10.7)	0.213
**∆ CRP** mg/L	1 (1–4)	2 (0–3)	0.562
**∆ urate** μmol/L	40 (−2.0–81.5)	40.5 (6.8–77.5)	0.954
**M12**			
**Weight loss since baseline,** kg	16.1 (10.5–22.8)	16.6 (9.7–22.9)	0.885
**Weight loss/baseline weight** %	16.3 (10.5–22.4)	15.7 (9.8–21.2)	0.853
**∆BMI** kg/m^2^	5.7 (3.6–8.5)	6.0 (3.7–8.2)	0.657
**∆ CRP** mg/L	2 (0–4)	2 (0–3)	0.952
**∆ urate** μmol/L	28 (−6.5–68.5)	35.5 (−3.3–58.8)	0.775

BMI = Body Mass Index, CRP = C- reactive protein

### Body weight and disease activity after 24 months

In total, 35 patients were examined at the 24-month visit. The median weight was 92.7 (IQR 85.7–100.7) kg and the median weight loss 7.4 (IQR 5.1–14.0) % of the baseline weight. Forty percent (*N* = 14/35) of the patients still had a weight loss of 10% or more compared to baseline, and 37.1% (*N* = 13/35) had a weight loss of 5–10%. All patients had however regained some weight since the 12-month visit, in median 6.3 (IQR 3.4–9.3) kg.

At 24 months the swollen/66 and tender/68 joints count, Leeds enthesitis index, CRP, HAQ, DAS28-CRP and DAPSA scores were still significantly lower compared to baseline. (Table 2 and Fig. 2) Criteria for MDA was met by 45.7% (16/35) at the 24 -month visit (*p* = 0.016, compared to baseline). There were however no significant differences in BSA, DLQI, BASFI and VAS global health, pain and fatigue between baseline and 24 months.

Subgroup analyses showed that the women demonstrated a greater reduction in VAS Global health (*p* = 0.007) and DAS28CRP (∆DAS28CRP) (*p* = 0.011) than the men, but there was no significant difference in the change in BMI, CRP, swollen/tender joints, DAPSA, BSA, HAQ between the sexes. Patients treatment with a bDMARD had a greater reduction in tender joints (∆tender/68; *p* = 0.029) and DAPSA (∆DAPSA; *p* = 0.048) compared to patients without a bDMARD at 24 months. No comparisons between PsA patients and controls could be made at the 24-month visit.

**Fig. 3:**
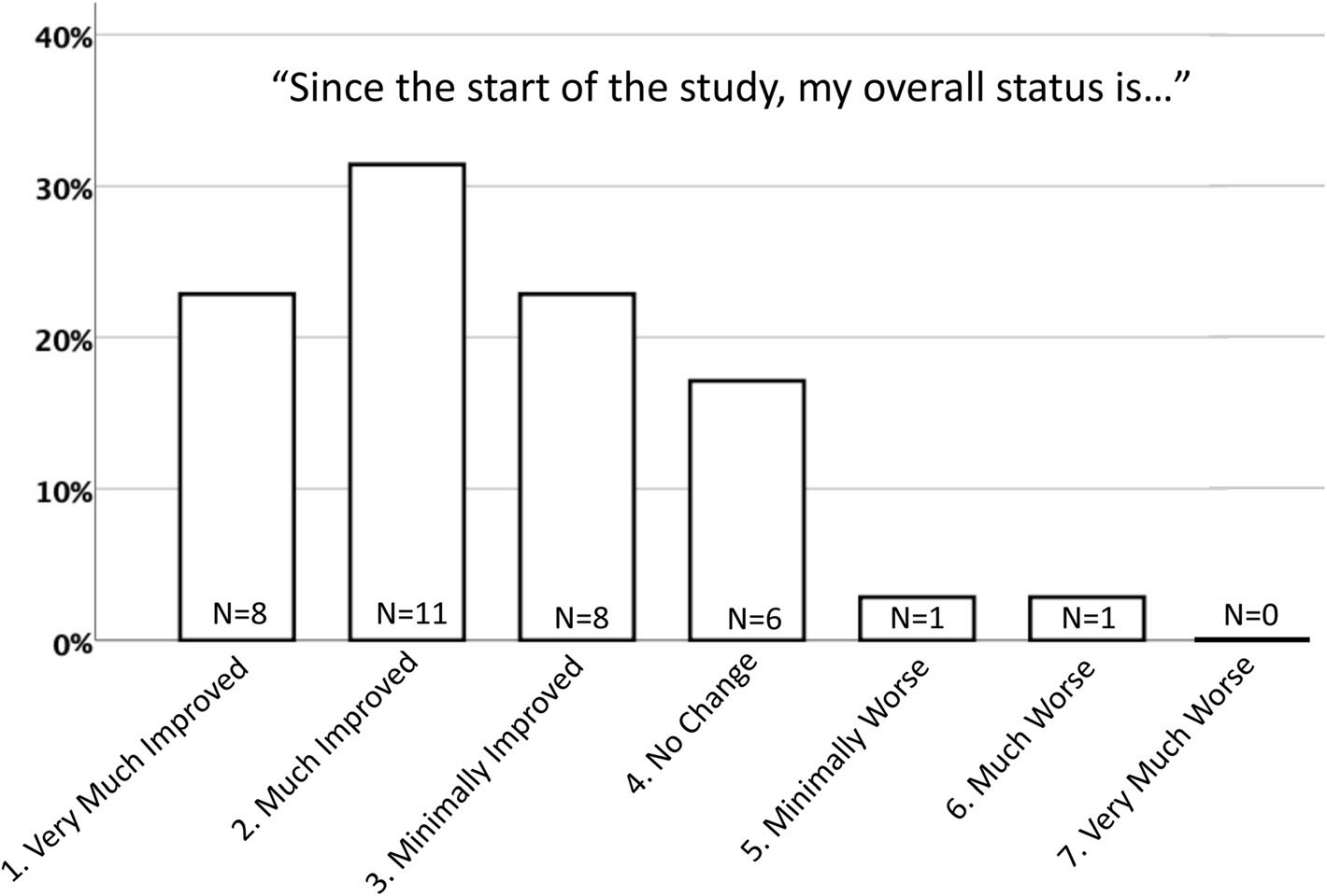
The Patient Global Impression of Change (PGIC) since the start of the study

Using PGIC to rate the PsA patients’ overall impression of change in status since study start, 54% of the patients reported “very much or much improved”, 23% “minimally improved”, 17% “no change” and 6% “worse”. (Fig. 3).

### Change in disease activity and self-reported function in relation to weight loss

**Fig. 4:**
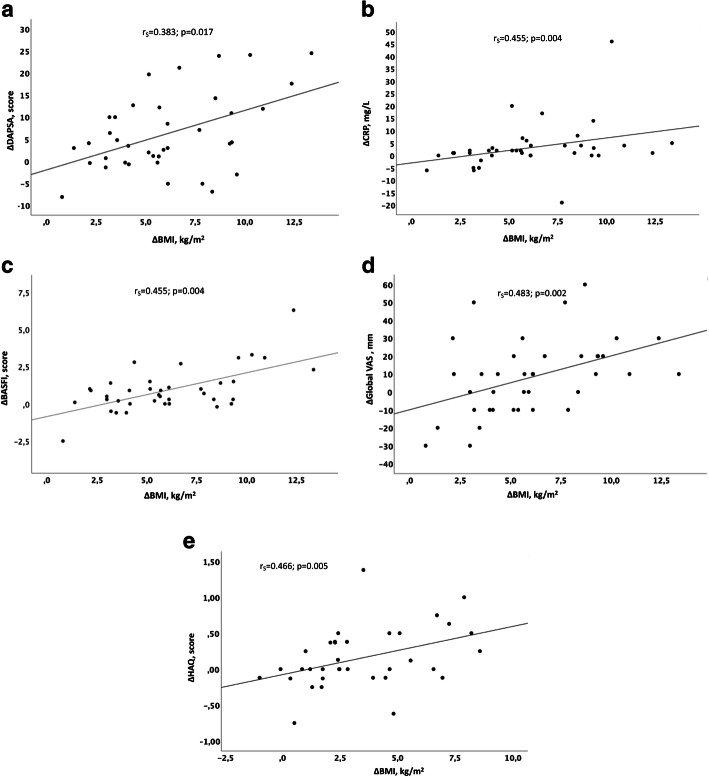
Scatterplots of the correlation between. **a** change in body mass index (ΔBMI) and change in Disease Activity in PSoriatic Arthritis score (ΔDAPSA) at the 12-month visit. **b** ΔBMI and change in C- reactive protein (ΔCRP) at the 12-month visit. **c** ΔBMI and change in Bath Ankylosing Spondylitis Functional Index (ΔBASFI) at the 12-month visit. **d** ΔBMI and change in Global Visual Analogue Scale (ΔGlobal VAS) at the 12-month visit. **e** ΔBMI and change in Health Assessment Questionnaire score (ΔHAQ) at the 24-month visit

At 12-months follow-up the change in BMI compared to baseline (∆BMI) was significantly correlated with ∆DAS28CRP (r_S_ = 0.526, *p* = 0.001), ∆DAPSA (r_S_ = 0.383, *p* = 0.017), ∆CRP (r_S_ = 0.455, *p* = 0.004), ∆BASFI (r_S_ = 0.455, p = 0.004) and ∆VAS for global health (r_S_ = 0.483, *p* = 0.002) in the PsA patients. (Fig. 4a-d) At 24-months follow-up ∆BMI was correlated with ∆HAQ (r_S_ = 0.466, *p* = 0.005) and ∆VAS for fatigue (r_S_ = 0.455, *p* = 0.006). (Fig. 4e) PGIC was also significantly associated with ∆BMI (r_S_ = − 0.412: *p* = 0.014) at 24 months.

### Change in variables associated with the metabolic syndrome

Data regarding the PsA patients is given in Table 5. At the six-month visit blood pressure, HbA1c and serum levels of TC, LDL, TG and glucose were significantly reduced, and HDL was significantly increased, compared to baseline. After 12-months there were still significant reductions in HbA1c, TG and glucose and an increase in HDL, and after 24-months a lowered TG and higher HDL remained. Criteria for MetS were met by 76.9% (30/39) at baseline, 35.9% (14/39) after six months, 38.5% (15/39) after 12-months, and by 60% (21/35) after 24-months.

**Table 5:** Parameters associated with the metabolic syndrome at baseline (BL) and after weight loss at 6 months (M6), 12 months (M12) and 24 months (M24) in patients with psoriatic arthritis (PsA) and obesity. Values are median and inter-quartile range (IQR)

	BL ***N*** = 39	M6 ***N*** = 39	M12 N = 39	M24 N = 35	BL-M6 p-value N = 39	BL-M12 ***p***-value N = 39	BL-M24 ***p***-value N = 35
**BMI** kg/m^2^	35.2 (34.0–37.8)	29.7 (26.2–31.4)	30.5 (28.0–33.0)	32.3 (30.2–35.4)	**< 0.001**	**< 0.001**	**< 0.001**
**Waistline** cm	115 (112–122)	95 (8–102)	97.5 (90–105)	107 (96–113)	**< 0.001**	**< 0.001**	**< 0.001**
**BP systolic** mmHg	127.5 (115–136)	120 (102.5–130)	121 (113.5–140)	124 (115–145)	**< 0.001**	0.410	0.701
**BP diastol** mmHg	77.5 (70–82.5)	70 (62.5–77.5)	72.5 (65–82.5)	75 (70–86)	**< 0.001**	0.073	0.891
**S-TC** mmol/L	5.5 (4.6–6.2)	4.9 (4.4–5.7)	5.4 (4.7–5.9)	5.6 (4.6–6.0)	**0.019**	0.508	0.227
**S-LDL** mmol/L	3.6 (3.0–4.4)	3.2 (2.6–3.9)	3.4 (2.8–4.0)	3.9 (2.8–4.5)	**0.003**	0.468	0.596
**S- HDL** mmol/L	1.4 (1.1–1.6)	1.4 (1.2–1.6)	1.65 (1.4–2.0)	1.6 (1.3–1.7)	**0.001**	**< 0.001**	**0.005**
**S- TG** mmol/L	1.6 (1.2–2.4)	1.1 (0.9–1.6)	1.2 (1.0–1.7)	1.4 (1.0–2.1)	**< 0.001**	**< 0.001**	**0.007**
**HbA1c** mmol/mol	35.5 (32.0–37.2)	32.5 (29.8–36.0)	34 (31–36)	35 (32–38)	**< 0.001**	**0.002**	0.203
**S-glucose** mmol/L	5.7 (5.3–6.2)	5.4 (5.1–5.8)	5.5 (5.0–5.9)	5.6 (5.2–6.2)	**< 0.001**	**0.006**	0.055
**Creatinine** μmol/L	75 (64–84)	70 (62–86)	72 (60–81)	74 (58–83)	0.066	**0.041**	**0.009**
**ALT** μkat/L	0.52 (0.41–0.68)	0.45 (0.36–0.62)	0.42 (0.31–0.55)	0.45 (0.31–0.58)	0.143	**0.015**	0.075
**Mets** n (%)	30 (76.9)	14 (35.9)	15 (38.5)	21 (60)	**< 0.001**	**< 0.001**	0.118

ALT = alanine transaminase, BMI = Body Mass Index, BP = blood pressure, HbA1c = glycosylated hemoglobin, HDL = high-density lipoprotein cholesterol, LDL = low-density lipoprotein cholesterol, S– =serum, TC = total cholesterol, TG = triglycerides, Mets = metabolic syndrome

The change in serum levels of TG (∆TG) was correlated with ∆DAPSA (r_S_ = 0.532, *p* = 0.002) and ∆DAS28CRP (r_S_ = 0.398, *p* = 0.027) at the 12 months visit. In addition, ∆LDL was correlated with ∆DAPSA (r_S_ = 0.437, p = 0.014) at the 24-month visit.

No significant differences were found for systolic and diastolic blood pressures at the 12- and 24-month visits compared to baseline, although three patients were able to stop treatment for hypertension during the study, and two patients could halve the dose of antihypertensives. Two patients stopped lipid-lowering therapy, while two other patients started lipid-lowering therapy during the follow-up. In addition, one patient started treatment with glucose-lowering medication during the study.

Data regarding the controls is given in supplementary Table 1. Similar to the PsA patients, the controls demonstrated lowered serum levels of TC, LDL, TG, HbA1c, S-glucose and ALT and increased serum levels of HDL. Criteria for MetS were met by 71.8% (28/39) of the controls at baseline, 48.7% (19/39) after six months, 53.8% (21/39) after 12-months.

Serum urate was also significantly reduced during the study in both PsA patients and controls. (Table 6). Baseline serum urate was similar in the PsA patients and controls, but significantly higher in men compared women in both the PsA patients (*p* = 0.002) and controls (*p* = 0.001). The decrease in serum urate (Δ-urate) was however not significantly correlated with Δ-BMI at any timepoint in the female and male patients and controls except in the PsA women at the 24-month visit (r_S_ = 0.493; *p* = 0.032).

**Table 6:** Serum urate in median and inter-quartile range (IQR) at baseline (BL) and after weight loss at 6 months (M6), 12 months (M12) and 24 months (M24) in women and men with psoriatic arthritis and obesity

S-urate μmol/L	BL	M6	M12	M24	BL-M6 p-value	BL-M12 p-value	BL-M24 p-value
**All patients**	330 (280–400) N = 39	301 (244–356) N = 39	306 (250–348) N = 39	324 (264–351) N = 35	**0.001**	**0.001**	**0.005**
**Women**	309 (269–361) *N* = 25	259 (232–350) N = 25	286 (242–348) N = 25	294 (234–338) *N* = 21	**0.023**	0.053	**0.033**
**Men**	394 (324–416) N = 14	324 (304–367) N = 14	319 (294–357) N = 14	334 (306–368) N = 14	**0.017**	**0.003**	0.064
**All controls**	350 (283–384) N = 39	280 (243–356) *N* = 39	306 (267–352) N = 39	N.A.	**< 0.001**	**0.001**	N.A.
**Women**	309 (278–366) *N* = 29	266 (230–299) N = 29	283 (235–321) N = 29	N.A.	**0.001**	**0.002**	N.A.
**Men**	400 (355–461) *N* = 10	359 (332–436) N = 10	372 (339–445) N = 10	N.A.	0.214	0.139	N.A.

## Discussion

In this prospective, open intervention study, we analyzed the long-term effects of weight loss on disease activity and self-reported physical function in patients with PsA and obesity. After 12 months of structured weight loss treatment, a median weight reduction of 16% was associated with improvement of a majority of disease activity measures compared to baseline, including swollen/tender joints count, enthesitis, extent of psoriasis, CRP, HAQ, BASFI and VAS global health and fatigue. After 24 months, all patients had regained some weight, but a significant improvement could still be shown in swollen/tender joints count, enthesitis, CRP and HAQ, compared to baseline. The results of the study support the hypothesis of obesity as a promotor of disease activity in PsA, showing what could be attained by adding a weight loss program to routine medical care in patients with PsA and obesity.

A secondary aim was to study the effects on variables associated with the MetS in PsA patients and matched controls. The magnitude of the weight loss was similar in the PsA patients and controls and associated with comparable reductions in waist circumference and serum levels of CRP, HBA1c, glucose, TC, LDL, TG and urate and increase in HDL, showing that the beneficial effects of weight loss treatment on inflammation and cardiovascular risk factors is general.

In the present study the weight loss treatment with VLED was judged as easy to implement by most PsA patients, but the transition from VLED to normal food was perceived as harder [20]. A substantial weight reduction was noted during the VLED treatment, but as expected the participants started to gain weight already at six months. The participants were given an energy-reduced dietary advice and were instructed to engage in health-enhancing physical activity at least 150 min per week, but no follow up was made between 12- and 24-month visits. The weight regain, especially between 12- and 24 months in the PsA patients, is a marker of a lowering of adherence to the energy intake restriction given during the first year. It is a well-documented challenge to keep weight off, even in structured weight loss programs [32, 33] Dansinger et al. found that self-reported dietary adherence was associated with 12-month weight change independent of macronutrient composition of the diet [34]. At the 24-month visit 40% of the PsA patients still had a weight reduction of 10% or more compared to baseline in the present study. Several studies in both specialist and primary care have included VLED as part of a structured weight loss program for subjects with severe obesity and have shown clinically meaningful weight loss, and the results of the present study are comparable with those [32, 33, 35–37]. Given the difficulties to treat obesity in a longer perspective once it is established, more attention should be given to prevent weight gain and the development of obesity in patients with psoriasis and PsA. We propose that the patients should be informed about the unfavorable effects of obesity on disease course and cardiovascular risk during patient educational programs and that body weight should be routinely measured and discussed during medical follow-ups. We also argue that patients with overweight should aim for weight maintenance and patients with obesity should be offered a weight loss program and be encouraged to participate in health-enhancing physical activity throughout the disease course.

Totally 11 PsA patients and 13 controls were lost to follow-up during the study. It is possible that the participants who did not come to follow-up had a poorer weight maintenance than the participants who continued in the study.

Studies on the effects of weight loss on disease activity in PsA are scarce, but there are a few studies on psoriasis. Effect on disease severity in psoriasis has been shown after weight loss by bariatric surgery [38, 39] and dietary interventions [40–43]. Improved response to cyclosporine [44] and TNFi [45] has also been shown in psoriasis after weight loss. Additionally, bariatric surgery in obesity has been associated with a lower future risk of developing psoriasis in one study [46], and psoriasis and PsA in another study [47]. One prior randomized controlled study on dietary interventions in patients with PsA and overweight or obesity starting treatment with TNFi demonstrated that a weight loss of ≥5% increased the chance of reaching MDA at six months follow-up [48]. The odds ratio (OR) for achieving MDA at a weight loss 5–10% and > 10% was 3.75 (95% CI 1.36–10.36) and 6.67 (95% CI 2.41–18.41) respectively.

Obesity is a pro-inflammatory state, where the adipose tissue is invaded by activated M1-type macrophages and B- and T-lymphocytes, with an over-production of cytokines, such as tumor necrosis factor-α (TNFα) and interleukin (IL)1, IL6, IL17 and IL23, which are all involved in the pathogenesis of psoriasis and PsA [49, 50]. Moreover, pro-inflammatory adipokines (resistin, fetuin-A, chemerin, leptin) are over-expressed in the adipocytes in obesity and adipokines with mainly anti-inflammatory effects (adiponektin, omentin) are supressed [51]. In the present study we show sustained lowered disease activity at the 12- and 24-month visits, when the patients were unlikely to be affected by the severe initial energy restriction from VLED, although the majority of participants had kept, to a different degree, an energy reduced diet. The anti-inflammatory effect of the weight loss may rather be explained by decreased production of pro-inflammatory cytokines and adipokines by the adipose tissue. Lowered mechanical loading by weight loss and less risk of microdamage, enthesitis and arthritis could also be explanatory factors.

In the present study, weight loss was associated with a reduction of other cardiovascular risk factors such as hypertension and dyslipidemia, and additionally lowered levels of blood glucose and HbA1c. MetS is defined as a set of cardiovascular risk factors that cluster together; enlarged waist circumference, high blood glucose levels, elevated blood pressure and TG, and reduced HDL [31]. Insulin resistance, central obesity and overabundance of circulating fatty acid are suggested mechanisms underlying the MetS [52]. The MetS is associated with increased mortality and poorer health outcomes in several aspects, such as a doubled risk of cardiovascular disease [53] and increased risk of various types of cancer [54]. In psoriasis and PsA there is a clear overrepresentation of cardiovascular risk factors and of MetS [4, 14, 55, 56] and the patients are at higher risk of developing cardiovascular events [5, 9]. The present study shows that treatment of obesity entails both reduction in disease activity, possibly through reduction in pro-inflammatory state, as well as cardiovascular risk factor. Although patients increased the energy intake and thereby gained in weight, they may have achieved some healthy food choices, for example reduced intake of sugary foods, and foods low in saturated fats and increased foods high in fiber and unsaturated fats that may improve cardiovascular risk factors. Unfortunately, we did not do any dietary assessments in the study.

Patients with psoriasis and PsA are at increased risk of developing gout [57]. The participants, both PsA patients and the controls, of the present study demonstrated significant decreases in serum urate. We could however not demonstrate any systematic correlation between the reduction in serum urate and the magnitude of weight loss, perhaps due to a small sample size and large interindividual weight change. Changes in dietary habits, including consumption of alcohol and sugar-sweetened soft drinks, may have played the key role in the reduction of serum urate.

### Strengths and limitations

Strengths of the study are the prospective design with a long follow-up and the powerful intervention which resulted in a considerable weight loss.

There are also limitations of the present study to be acknowledged. Firstly, this is not a randomized controlled study and the absence of an untreated control group with PsA is a major short-coming. A control group consisting of patients with obesity, but otherwise healthy, matched for sex, age and bodyweight, undergoing the same VLED treatment was however included in the study, to enable comparisons of vital signs and biochemical measures. Secondly, a threshold for disease activity was not among the inclusion criteria of the study. The study included patient from every day practice, whereof some fulfilled criteria for MDA already at baseline and some were near fulfillment of MDA. The data concerning MDA should therefor be interpreted with caution, since it is possible that even a minor improvement can have turned some of the non-MDA patients into MDA. In addition, the number of included patients was small. Nevertheless, the study was able to show significant association between weight loss and reduced disease activity.

Thirdly, treatment with cs/bDMARDs was held constant from three month prior to baseline until the six-month visit, but change in the cs/bDMARD treatment was allowed after the six-month visit, due to ethical reasons. We cannot exclude that change in treatment may have affected the results regarding disease activity at the 12- and 24-month visits. In the vast majority of patients, the medication with cs/bDMARDs was however held unchanged during the whole study period. Fourthly, pharmacological treatment against hypertension, hyperlipidemia and diabetes could not be held constant throughout the study, which may have affected levels of blood pressure, blood lipids and glucose metabolism during the follow-up. During the initial period with intense weight loss, some patients experienced hypotension and antihypertensive treatment had to be tapered. Fifthly, there were no treatment or follow-up visits during the second year that also may have affected the weight regain among most PsA patients. The results indicate that prolonged treatment periods for weight maintenance or further weight loss may be needed.

## Conclusions

Weight loss treatment with VLED in patients with PsA and obesity was associated with sustained lowered disease activity after 12 months and 24 months of follow-up, with concurrent improvements in cardiovascular risk factors and lowered serum urate. The results of the study provide support to the hypothesis of obesity as a promotor of disease activity in PsA and shows what can be achieved by 5–15% weight loss in patients with PsA and obesity.

## Supplementary information

**Additional file 1: Suppl Table 1.** Parameters associated with the metabolic syndrome at baseline (BL) and after weight loss at 6 months (M6) and 12 months (M12) in controls with obesity. Values are median and inter-quartile range (IQR).

## Data Availability

The datasets analyzed during the current study are available from the corresponding author on reasonable request.

## Abbreviations

ACR: American College of Rheumatology
ALT: alanine transaminase
BASFI: Bath Ankylosing Spondylitis Functional Index
BMI: Body Mass Index
BSA: Body Surface Area
CRP: C- reactive protein
CASPAR: Classification for Psoriatic Arthritis
CV: cardiovascular
DAPSA: Disease Activity in PSoriatic Arthritis
DAS28CRP: Disease Activity Score using 28 joint counts based on CRP
DLQI: Dermatology Life Quality Index
cs/b DMARD: conventional synthetic / biologic Disease Modifying Anti-Rheumatic Drug
ESR: erythrocyte sedimentation rate
HAQ: Health Assessment Questionnaire
Hb: hemoglobin
HbA1c: glycosylated hemoglobin
HDL: high-density lipoprotein cholesterol
IL: interleukin
IQR: interquartile range
LDL: low-density lipoprotein cholesterol
MDA: Minimal Disease Activity
MetS: metabolic syndrome
NSAID: non-steroidal anti-inflammatory drug
PLT: platelet count
PsA: psoriatic arthritis
r_S_: Spearman’s correlation
TC: total cholesterol
TG: triglycerides
TNFα: Tumor necrosis factor α
TNFi: tumor necrosis factor inhibitor
VAS: Visual Analogue Scale
VLED: Very Low Energy Diet
WBC: White Blood Cell count
