# Supplementary material for: Weight loss is associated with sustained improvement of disease activity and cardiovascular risk factors in patients with psoriatic arthritis and obesity: a prospective intervention study with two years of follow-up
Source: Arthritis Res Ther. 2020 Oct 22;22:254. doi: 10.1186/s13075-020-02350-5 (PMC7583178; doi:10.1186/s13075-020-02350-5)
Supplement: Supplementary file 1 — Additional file 1: Suppl Table 1. Parameters associated with the metabolic syndrome at baseline (BL) and after weight loss at 6 months (M6) and 12 months (M12) in controls with obesity. Values are median and inter-quartile range (IQR). [file 13075_2020_2350_MOESM1_ESM.docx]

**Suppl table 1** Parameters associated with the metabolic syndrome at baseline (BL) and after weight loss at 6 months (M6) and 12 months (M12) and 24 in controls with obesity. Values are median and inter-quartile range (IQR).

|  | **BL**  **N=39** | **M6**  **N=39** | **M12**  **N=39** | **BL-M6**  **p-value**  **N=39** | **BL-M12**  **p-value**  **N=39** |
| --- | --- | --- | --- | --- | --- |
| **Body weight**  kg | 107  (96.5–122.2) | 80.5  (76.0–94.0) | 87.6  (78.3–97.5) | **<0.001** | **<0.001** |
| **BMI**  kg/m^2^ | 38.5  (37.0–41.7) | 30.5  (28.0–34.0) | 32.6  (30.3–34.8) | **<0.001** | **<0.001** |
| **Waistline**  cm | 117.0  (107–126.5) | 93.5  (86.8–105.0) | 100.0  (92.0–109.0) | **<0.001** | **<0.001** |
| **BP systolic**  mm Hg | 122  (115–137) | 127  (115–137) | 124  (118–139) | 0.342 | 0.194 |
| **BP diastolic**  mm Hg | 77  (70–85) | 74  (69–81) | 78  (68–85) | 0.192 | 0.700 |
| **CRP**  mg/L | 4  (2–6) | 2  (1–4) | 1  (1–3) | **<0.001** | **<0.001** |
| **Hb**  g/L | 141  (135–149) | 139  (133–147) | 140  (132–148) | 0.059 | 0.477 |
| **WBC**  10^9^/L | 6.6  (5.6–7.6) | 6.0  (4.9–6.4) | 5.5  (4.6–6.8) | **0.003** | **<0.001** |
| **PLT**  10^9^/L | 266  (225–302) | 248  (204–284) | 264  (219–319) | **0.026** | 0.230 |
| **S-TC**  mmol/L | 4.9  (4.1–5.6) | 4.6  (3.7–5.4) | 4.7  (3.9–5.6) | **0.013** | 0.141 |
| **S-LDL**  mmol/L | 3.2  (2.5­4.0) | 3.1  (2.2–3.9) | 2.8  (2.2–3.8) | 0.059 | 0.081 |
| **S- HDL**  mmol/L | 1.4  (1.0–1.6) | 1.4  (1.2–1.7) | 1.6  (1.2–1.9) | **0.018** | **<0.001** |
| **S- TG**  mmol/L | 1.7  (1.2–2.1) | 0.97  (0.74–1.3) | 1.1  (0.79–1.6) | **<0.001** | **<0.001** |
| **HbA1c** mmol/mol | ∆ | 33  (31–36) | 35  (32–38) | **<0.001** | **<0.001** |
| **S-glucose**  mmol/L | 6.2  (5.8–7.1) | 5.6  (5.0–6.0) | 5.7  (5.2–6.6) | **<0.001** | **<0.001** |
| **Creatinine**  µmol/L | 68  (62–79) | 65  (58–76) | 70  (61–75.5) | **0.017** | 0.747 |
| **S-urate**  µmol/L | 350  (283–384) | 280  (243–356) | 306  (267–352) | **<0.001** | **0.001** |
| **ALT**  µkat/L | 0.50  (0.42–0.68) | 0.41  (0.31–0.48) | 0.40  (0.35–0.52) | **<0.001** | **0.002** |
| **Mets**  n (%) | 28  (71.8) | 19  (48.7) | 21  (53.8) | **0.004** | **0.039** |

ALT = alanine transaminase, BMI = Body Mass Index, BP = blood pressure, CRP = C- reactive protein, Hb = haemoglobin HbA1c = glycosylated hemoglobin, HDL = high-density lipoprotein cholesterol, LDL = low-density lipoprotein cholesterol, Mets = metabolic syndrome, PLT = platelet count, S– =serum, TC = total cholesterol, TG = triglycerides, WBC = White Blood Cell count
